# Vaccinia and Smallpox

**Published:** 1901-05-18

**Authors:** 


### VACCINIA AND SMALLPOX.

There may be a few people who still believe that cow-pox is an entirely independent disease, the occurrence of which, accidentally so to say, and for some inexplicable reason happens to afford protection from smallpox. The great mass of those who have thought about or inquired into the subject believe, however, that vaccinia is but a modification of smallpox, arising in some way from its passage through the cow. It is only by regarding the matter in this light that the protection afforded by vaccination falls into line with what we know about the operation of other forms of infection, and about the manner in which protection against other forms of infective disease may be obtained by inoculation with attenuations of their germs. Yet there has been a standing difficulty in the way of accepting this view. If vaccinia were but the bovine form of smallpox it ought surely to be possible to produce it by inoculating smallpox upon the cow; and the great difficulty which has always been found in transmitting human smallpox to bovines, whether cows or calves, has not infrequently been cited as a reason for regarding with distrust the theory first expounded by Jenner, and later held with various modifications by most observers, that cow-pox as met with in the cow and among milkers was originally derived from smallpox in the human body. It has to be remembered, however, that in the days when cow-pox was so common among cows that milkers often became infected with it, and thus obtained protection against smallpox, the type of disease from which these cows would be likely to be infected would be the ambulant form. A great deal at any rate of the smallpox which was prevalent during the time that Jenner lived and wrote was of that comparatively mild variety which, under the name of inoculated smallpox, was intentionally produced in healthy subjects with the object of conferring protection against the more virulent forms of the malady. Turning this matter over in his mind Dr. Monckton Copeman[1] came to the conclusion that it was not improbably from this inoculated form of smallpox rather than from the ordinary form that much of the cow-pox in the prevaccination period was derived. Being unable to obtain for experiment matter from smallpox inoculated in the human subject, Dr. Copeman had recourse to monkeys, which are readily susceptible to the disease, and in which the various phases of the disease closely resemble those observed in man. Smallpox material was obtained from cases at Middlesbrough, London, and Glasgow, and in each of three series of experiments the lymph was, as a first step, inoculated directly on calves, in every instance with altogether negative results. But with monkeys success was as invariably obtained, and when after one or more passages through these animals the contents of the inoculation vesicles were employed for insertion on the calf an effect was now produced which, after one or more removes in this animal, was indistinguishable from typical vaccinia. From these vesicles a number of children were vaccinated and afterwards kept under observation for a couple of months. Every such vaccination "took" normally, and in no case was any bad result or any generalisation of the eruption observed. The point of interest in this experiment lies in the fact that whereas the human smallpox material employed could not be got to "take" directly on the calf, nevertheless results typical of ordinary vaccinia were obtained when the same strain of lymph, after passing through a series of monkeys, was again transferred to the epidermis of the calf. Thus we have another proof that vaccinia is but a modification of smallpox, and that the protection afforded by it falls into line with other forms of protection obtained by the "attenuation" of living virus of one form and another.

[1] Brit. Med. Jour., May 11.

